# SARS-CoV-2-specific circulating T follicular helper cells correlate with neutralizing antibodies and increase during early convalescence

**DOI:** 10.1371/journal.ppat.1009761

**Published:** 2021-07-16

**Authors:** Sushma Boppana, Kai Qin, Jacob K. Files, Ronnie M. Russell, Regina Stoltz, Frederic Bibollet-Ruche, Anju Bansal, Nathan Erdmann, Beatrice H. Hahn, Paul A. Goepfert

**Affiliations:** 1 Division of Infectious Diseases, Department of Medicine, University of Alabama at Birmingham, Birmingham, Alabama, United States of America; 2 Department of Medicine, University of Pennsylvania, Philadelphia, Pennsylvania, United States of America; 3 Department of Microbiology, University of Pennsylvania, Philadelphia, Pennsylvania, United States of America; St. Jude Children’s Research Hospital, UNITED STATES

## Abstract

T-cell immunity is likely to play a role in protection against SARS-CoV-2 by helping generate neutralizing antibodies. We longitudinally studied CD4 T-cell responses to the M, N, and S structural proteins of SARS-CoV-2 in 26 convalescent individuals. Within the first two months following symptom onset, a majority of individuals (81%) mounted at least one CD4 T-cell response, and 48% of individuals mounted detectable SARS-CoV-2-specific circulating T follicular helper cells (cTfh, defined as CXCR5^+^PD1^+^ CD4 T cells). SARS-CoV-2-specific cTfh responses across all three protein specificities correlated with antibody neutralization with the strongest correlation observed for S protein-specific responses. When examined over time, cTfh responses, particularly to the M protein, increased in convalescence, and robust cTfh responses with magnitudes greater than 5% were detected at the second convalescent visit, a median of 38 days post-symptom onset. CD4 T-cell responses declined but persisted at low magnitudes three months and six months after symptom onset. These data deepen our understanding of antigen-specific cTfh responses in SARS-CoV-2 infection, suggesting that in addition to S protein, M and N protein-specific cTfh may also assist in the development of neutralizing antibodies and that cTfh response formation may be delayed in SARS-CoV-2 infection.

## Author summary

Since December 2019, the Coronavirus Disease 2019 (COVID-19) pandemic has caused significant morbidity and mortality worldwide. Recently approved vaccines against SARS-CoV-2 are understood to protect against infection by inducing neutralizing antibodies. However, the underlying immune responses necessary for protection remain unclear. It is well established that T follicular helper cells (Tfh), a subset of CD4 T cells, are essential to the development of neutralizing antibodies and that some of these cells, called circulating T follicular helper cells (cTfh), can be studied in the blood. Not much is known about Tfh responses mounted in SARS-CoV-2 infection. Here, we studied cTfh responses to three major structural proteins in individuals recovered from COVID-19. We find that SARS-CoV-2-specific cTfh frequencies correlate with neutralizing antibody responses. We also find that cTfh responses to SARS-CoV-2 increase well into convalescence before contracting. Our results suggest that cTfh responses against proteins other than the spike protein may contribute to the development of neutralizing antibodies and that the formation of cTfh responses in SARS-CoV-2 infection may be delayed.

## Introduction

Cases of COVID-19, caused by the novel severe acute respiratory syndrome coronavirus 2 (SARS-CoV-2), were first reported in Wuhan, China at the end of 2019 [1]. Since then, the COVID-19 pandemic has caused significant morbidity, mortality, and economic disruption worldwide [2]. In SARS-CoV-2 infection, initial studies reported significant lymphopenia in hospitalized patients [3]. Data on antigen-specific T-cell responses in individuals recovered from SARS-CoV-2 infection have reported CD4 T-cell responses to SARS-CoV-2 in 80–100% of convalescent individuals, with most publications focusing on the spike (S) protein [4–7]. Other groups have shown that T cells also target the nucleocapsid (N) and membrane (M) proteins [8].

The approval of effective SARS-CoV-2 vaccines has been heralded as a critical step to curtailing the COVID-19 pandemic. Studies in non-human primates have found that neutralizing antibodies (nAb) are a correlate of protection in infection and vaccination [9,10]. Phase I/II trial data from the Pfizer and Moderna vaccines, as well other candidate vaccines, have highlighted neutralizing antibodies as the driving force of vaccine efficacy [11–14]. While the Pfizer vaccine has been shown to elicit CD4 and CD8 T-cell responses in trial participants and that vaccine-induced CD4 T-cell responses correlated with antibody titers [15], our understanding of exact immune correlates of protection and the durability of these immune responses remains incomplete. Additionally, the factors driving the formation of nAb in the context of SARS-CoV-2 natural infection are important to study, particularly in the context of emerging viral variants that could be differentially infectious [16,17]. In both mild and severe SARS-CoV-2 disease, many groups have described an elevation of both activation and exhaustion markers on T cells [18–21]. Our group has demonstrated that SARS-CoV-2-induced immune dysregulation persists into convalescence [20].

Because direct study of lymphoid tissues in humans is difficult, circulating T follicular cells (cTfh), or T follicular helper cells (Tfh) circulating in the blood, serve as an important surrogate for understanding Tfh responses within germinal centers. While there is some controversy regarding how to best identify these cells, there is general consensus that these cells express CXCR5, a lymph node homing receptor, and many groups use PD1 expression in conjunction with CXCR5 to define cTfh [22–24]. While frequencies of circulating CXCR5^+^PD1^+^ CD4 T cells are typically low, these cells are closely linked to Tfh in lymphoid tissue [25] and have been shown to support humoral responses [26,27]. Antigen-specific cTfh have been shown to correlate with neutralizing antibodies in the context of infection and vaccination of several pathogens [23,28–32]. Although cTfh responses have not been described in the context of SARS-CoV or MERS-CoV infection, CD4 T-cell responses have been shown to be important in controlling SARS-CoV in mouse models [33], and a recent study of a MERS-CoV vaccine in mice found that Tfh frequencies in draining lymph nodes correlated with neutralizing antibodies [34].

There is an increasing amount of data on SARS-CoV-2-specific T follicular helper cell responses. Thevarajan et al. were the first to report cTfh frequencies in SARS-CoV-2 and found that frequencies of total cTfh increased during acute infection [35]. Since then, a few studies have drawn a correlation between total CD4 T-cell or total Tfh-like cell frequencies and antibody levels [36,37]. One study found increased expression of CXCR5 and ICOS, two Tfh markers, on SARS-CoV-2-specific CD4 T-cells but did not examine cTfh responses directly [38]. In deceased donors with COVID-19, Kaneko et al. found that BCL6-expression in germinal center Tfh was lost within thoracic lymph nodes, suggesting that Tfh response formation may be impaired in severe SARS-CoV-2 infection [39], but the formation of antigen-specific Tfh responses, particularly in milder cases of COVID-19, remains unclear.

Juno et al examined circulating Tfh, defined as CD45RA^-^CXCR5^+^ CD4 T cells, in the blood of SARS-CoV-2 infected individuals. They demonstrated a correlation between S protein-specific cTfh and nAb, suggesting that functional Tfh responses are formed in mild SARS-CoV-2 infection [40]. However, these data leave several questions unanswered, including at what point in convalescence these responses evolve. While this study was a useful first glimpse at antigen-specific Tfh responses and included PD1, a canonical Tfh marker, in their panel, PD1 expression was not used to define the Tfh population and was not reported. Additionally, the study used Ox40 and CD25 as activation markers to identify antigen-specific responses, which have previously been shown to include a high percentage of T regulatory cells [41]. Another recent study examined the persistence of S protein-specific CD4 T-cell responses in convalescence [42]. They examined frequencies of circulating Tfh (ICOS^+^CXCR5^+^ CD4 T cells) roughly one month and three months after symptom onset, and although they saw responses greater than background at three months, they did not see a difference over time. A more detailed examination of CD4 T-cell and cTfh responses in convalescence would help to establish how these responses develop over time. More recent studies have investigated cTfh populations up to 6 months after initial symptom onset [43,44]. However, these longitudinal studies did not investigate correlations between antigen-specific cTfh and SARS-CoV-2-specfic antibodies. It is also important to note that all of these studies primarily focused on S protein-specific responses. cTfh specificity does not necessarily correspond with neutralizing antibody specificity. For example, in HIV infection and vaccination, intrastructural help occurs, where CD4 T-cell responses to internal, structural proteins correlate with neutralizing antibodies against the exterior, envelope protein [45,46], underscoring the importance of examining cTfh responses across the SARS-CoV-2 proteome.

Here, we report on SARS-CoV-2-specific CD4 T-cell responses to the membrane (M), nucleocapsid (N), and spike (S) proteins studied longitudinally in 26 convalescent individuals. We directly examined antigen-specific cTfh (CXCR5^+^PD1^+^ CD4 T cells) and observed correlations between antigen-specific cTfh responses across all protein specificities and antibody neutralization at the first convalescent visit. We find the M protein-specific cTfh responses increase in magnitude from Visit 1 to Visit 2. High magnitude SARS-CoV-2-specific cTfh responses (>5% activation of total cTfh population) were only detected at the second convalescent visit, more than 30 days following symptom onset, but these responses do not correlate with antibody neutralization. These data are the first to examine the kinetics of cTfh responses that arise after SARS-CoV-2 infection as well as the relationship between neutralizing antibodies and cTfh responses to the SARS-CoV-2 M and N proteins. Our findings also suggest that cTfh formation may be delayed in SARS-CoV-2 infection.

## Results

### SARS-CoV-2-specific CD4 T cells targeting the M, N, and S proteins are detected in individuals recovered from COVID-19 at their first convalescent visit

In 26 individuals recovered from COVID-19, we assessed the presence of T-cell responses to the membrane (M), nucleocapsid (N), and spike (S) proteins of SARS-CoV-2 using overlapping 20mer peptide pools spanning each protein. All but two of these individuals were confirmed to have SARS-CoV-2 infection by PCR, and the two who were not PCR tested reported a known COVID-19 contact and had detectable SARS-CoV-2-specific T-cell responses. While none of these individuals required hospitalization, all experienced COVID-19 related symptoms, and a majority (62%) reported a moderate severity of symptoms. T-cell responses were measured at the first convalescent visit for each individual, which occurred a median of 22 days post-symptom onset, and at the second visit, which was a median of 38 days post-symptom onset (**Table 1**). We utilized two flow cytometry-based strategies: 1) upregulation of activation-induced markers (AIM) and 2) production of effector molecules (IFNγ, TNFα, CD154) by intracellular cytokine staining (ICS). 26 individuals were assessed by AIM, and 21 of these individuals were assessed in parallel by ICS. Gating strategies for AIM and ICS in an unstimulated, negative control are shown in **S1 Fig**.

**Table 1:** Patient demographics.

	Convalescent (N = 26)	Healthy Control (N = 10)
**Age**	41 (20, 76)	41 (30, 50)
**Sex**		
** Female**	38% (10/26)	60% (6/10)
** Male**	62% (16/26)	40% (4/10)
Days post-symptom onset*		
** Visit 1**	22 (12, 40)	NA
** Visit 2**	38 (26, 59)	NA
** Visit 3** (N = 4)	98 (94, 105)	NA
** Visit 4** (N = 3)	192 (185, 196)	NA
Days between visits*	14 (5, 27)	NA
**Symptom severity**		
** Mild (1)**	38% (10/26)	NA
** Moderate (2)**	62% (16/26)	NA
** Severe (3)**	0% (0/26)	NA

*Values reported as median with range in parentheses. NA signifies the data is not available.

**Fig 1:**
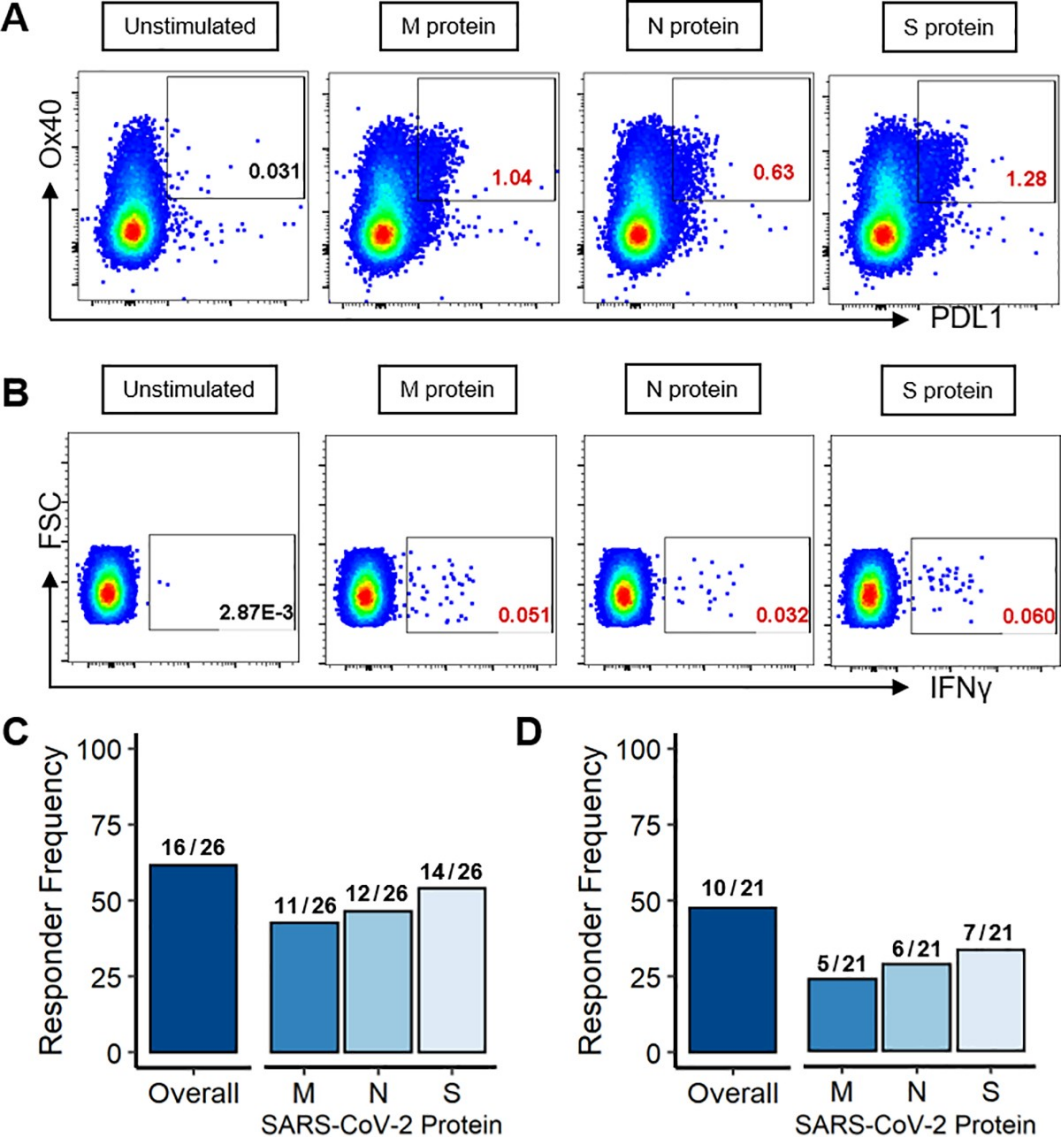
SARS-CoV-2-specific CD4 T cells target the M, N, and S proteins in individuals recovered from COVID-19 at their first convalescent visit. Representative examples of CD4 T-cell responses in CID8 to the M, N, and S protein peptide pools as detected by upregulation of activation-induced markers, Ox40 and PDL1 **(A)** and by IFNγ in intracellular cytokine staining **(B)**. Responder frequency of CD4 T-cell responses to any SARS-CoV-2 protein and to the M, N, and S proteins individually by AIM **(C,** N = 26**)** and ICS **(D,** N = 21**)**. Positive SARS-CoV-2-specific responses are indicated by gate frequencies in red.

Representative positive CD4 T-cell responses measured by each staining strategy are shown in **Fig 1A** for AIM and in **Fig 1B** for ICS in one convalescent individual, CID8, who mounted CD4 T-cell responses against all three SARS-CoV-2 proteins. At the first convalescent visit, we found that 62% (16/26) of individuals mounted a SARS-CoV-2-specific CD4 T-cell response by AIM and that these CD4 responses targeted all three tested proteins (**Fig 1C**). Meanwhile, by ICS, 47% (10/21) of individuals had at least one SARS-CoV-2-specific CD4 response, with a similar distribution across the tested proteins (**Fig 1D**). As a control, we also measured T-cell responses to SARS-CoV-2 peptide pools in COVID-19 negative individuals by assaying samples collected from healthy individuals before the COVID-19 pandemic. In the healthy controls tested, we detected three low magnitude (≤ 0.17%), presumably cross-reactive memory, CD4 T-cell responses in two of the ten tested individuals (20%) in line with previously published reports [5]. Representative staining of an AIM-detected and an ICS-detected response in the COVID negative controls is shown in **S2A and S2B Fig**, with overall responder frequencies in **S2C and S2D Fig**. Overall, our data show that a majority of convalescent individuals mounted a SARS-CoV-2-specific CD4 T-cell response as detected by activation marker expression and/or cytokine production and that these responses targeted all three structural proteins assessed.

In the 21 individuals assessed by both AIM and ICS, there was a weak but significant correlation between the response magnitude for AIM and ICS for each condition (p = 0.004, r = 0.33; **S3 Fig**). However, more responses were identified and there was an increased responder frequency by upregulation of activation-induced marker expression than by intracellular cytokine staining (**Fig 1**), as have been shown by previous groups [41,47]. There was high agreement between CD4 responses by AIM and ICS assays, with 17 of the 18 positive ICS responses (94%) having a corresponding positive AIM response. These data also show that 12 CD4 responses detected by AIM in early convalescence were not detected by our ICS assay investigating IFNγ, TNFα, or CD154. This suggests that the functionality of these CD4 responses lies outside of the assayed cytokines and highlights the increased sensitivity of AIM at detecting antigen-specific CD4 T-cell responses. These results are similar to previous reports in convalescent COVID-19 subjects that have shown a 10-fold increase in activation-induced marker upregulation by AIM when compared to cytokine production by ICS [48].

### SARS-CoV-2-specific circulating T follicular helper cells are detected in convalescent individuals

**Fig 2:**
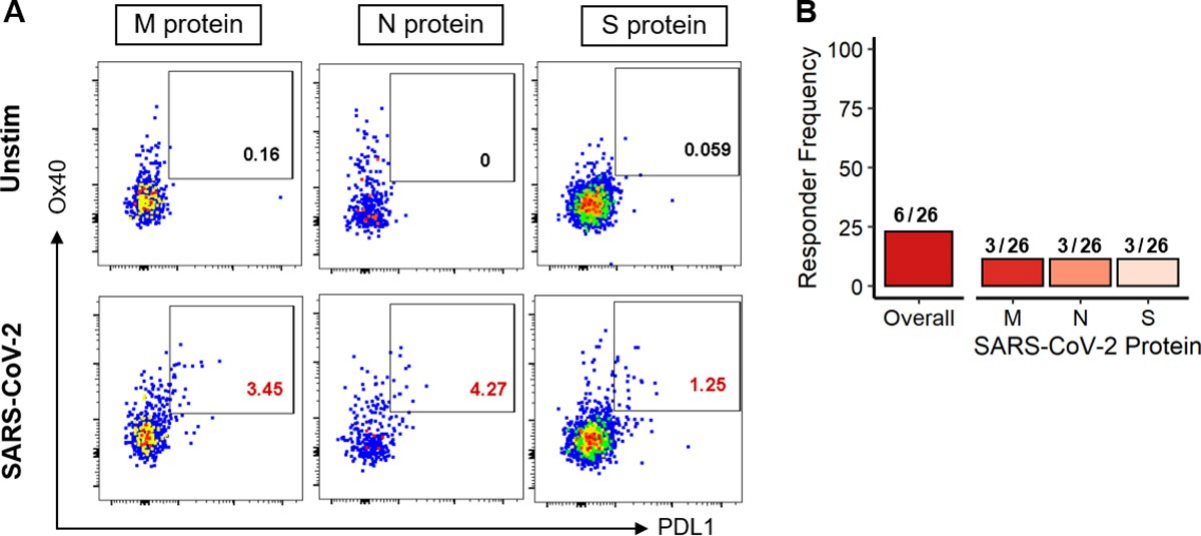
SARS-CoV-2-specific circulating T follicular helper cells are detected at the first visit in 6 out of 26 convalescent individuals. **(A)** Representative examples of antigen-specific cTfh (CD4^+^CXCR5^+^PD1^+^) detected upon stimulation with SARS-CoV-2 M, N, and S protein peptides for Visit 1 in three individuals (CID8, CID11, and CID13, respectively). Negative control of unstimulated cells shown in the top row. **(B)** Frequency of individuals mounting a positive cTfh response at their first visit to any SARS-CoV-2 protein and to the M, N, and S protein peptide pools.

We directly measured antigen-specific cTfh responses by the expression of Ox40 and PDL1 on CXCR5^+^PD1^+^ CD4 T cells (gating strategy shown in **S1 Fig**). Representative examples of SARS-CoV-2-specific cTfh responses to the M, N, and S proteins are shown in **Fig 2A** across three individuals, and all positive cTfh responses detected at Visit 1 are shown in **S4 Fig**. At the first convalescent visit, occurring a median of 22 days post-symptom onset, we detected 9 total cTfh responses in 6 of the 26 individuals tested (23%), equally spread across each of the three proteins (**Fig 2B**). These data indicate that a minority of individuals mounted detectable SARS-CoV-2-specific cTfh responses early in convalescence.

Meanwhile, none of the healthy controls tested had detectable SARS-CoV-2-specific cTfh responses. This lack of cTfh responses in COVID-19 negative individuals is not surprising, as cTfh compose a minor population of the total CD4 T cells in the blood, and cTfh responses induced by other seasonal coronaviruses, if present, likely exist at very low, undetectable frequencies. Additionally, the fact that these responses were only detected in convalescent individuals bolsters our confidence that these cTfh responses were definitively induced by recent SARS-CoV-2 infection.

### SARS-CoV-2-specific cTfh frequencies across the M, N, and S proteins correlate with antibody neutralization

**Fig 3:**
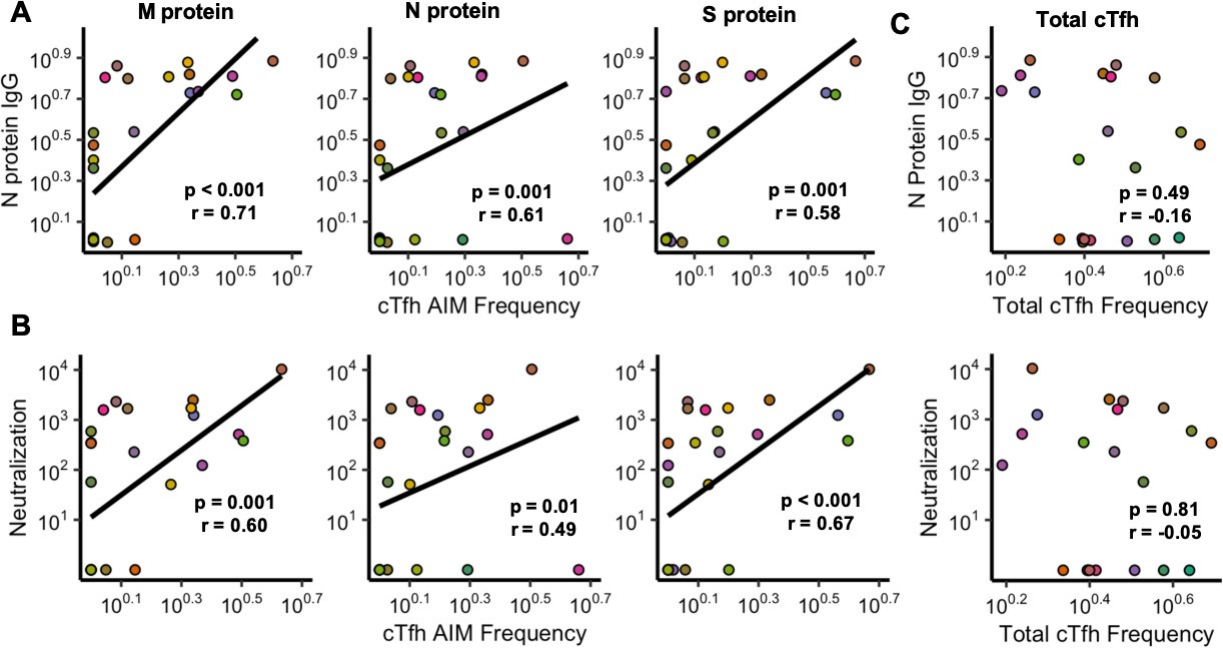
SARS-CoV-2-specific cTfh frequencies across the M, N, and S proteins correlate with antibody neutralization (N = 26). **(A)** Correlations between N protein IgG titers and cTfh frequencies towards the M, N, and S proteins. **(B)** Correlations between antibody neutralization (ID50, dilution of plasma at which luminescence was reduced to 50%) and cTfh frequencies. **(C)** Correlations between the total cTfh frequency and antibody titer and neutralization. (All correlations represented by a linear regression line. Axes are transformed by log10(x+1) to allow for visualization of 0s. Statistics were determined by a Spearman Correlation test. Points are colored for each individual).

Because cTfh are important for the development of an antibody response, we investigated whether the frequency of SARS-CoV-2-specific cTfh correlated with antibody level and neutralization at the first convalescent visit. We used two measurements of antibodies: The first was the commercially available Abbott test that detects N protein-specific IgG titers. The second assay measured antibody neutralization using an HIV-1 based pseudotyping assay, which is likely a more biologically relevant metric because neutralizing antibodies are thought to represent a key correlate of protection [9–12]. For all three proteins, we see a similar level of significance in the correlation between antigen-specific cTfh frequency and N protein IgG titer (**Fig 3A**). However, we find that cTfh frequencies across proteins differentially correlate with antibody neutralization (**Fig 3B)**: S protein-specific cTfh responses most strongly correlate with nAb (p < 0.0001, r = 0.66), followed by M protein-specific ones (p = 0.001, r = 0.60), and finally N protein-specific cTfh (p = 0.01, r = 0.50). To ensure these correlations were specific to SARS-CoV-2-induced responses, we also quantified the frequency of total cTfh (CXCR5^+^PD1^+^ CD4 T cells). No significant correlation between the overall frequency of cTfh and antibody levels or neutralization was observed (**Fig 3C**). Taken together, these data suggest that antigen-specific cTfh responses across SARS-CoV-2 proteins contribute to the development of more potent nAbs.

### SARS-CoV-2-specific circulating T follicular helper cell responses increase from Visit 1 to Visit 2

**Fig 4:**
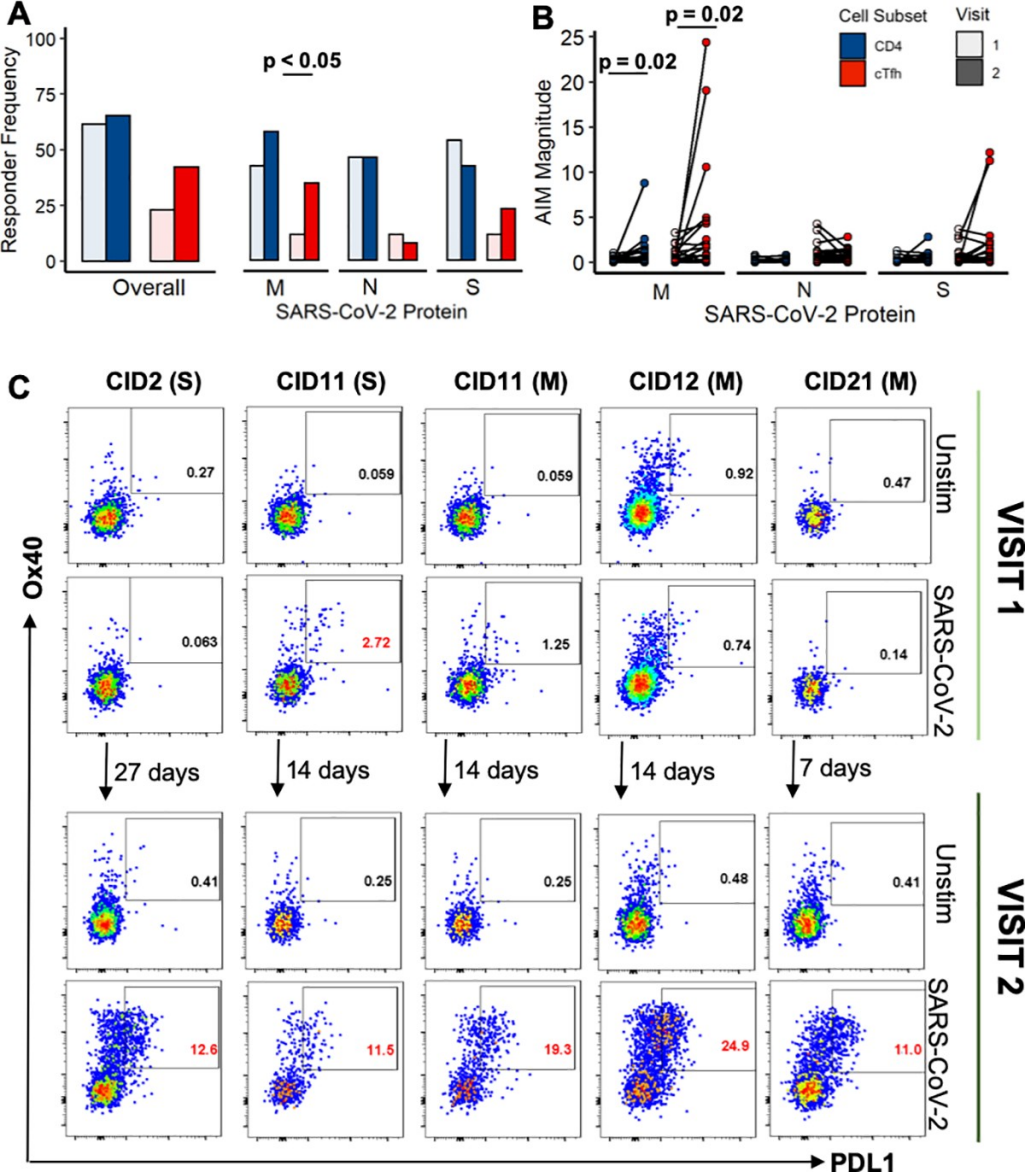
M protein-specific cTfh responses increase from the first to the second convalescent visit (N = 26). **(A)** Paired convalescence Visit 1 and Visit 2 CD4 T-cell and cTfh response magnitudes by AIM (p values determined by Fisher’s Exact Test). **(B)** Paired CD4 T-cell and cTfh response magnitudes for AIM (p values determined by a paired Wilcoxon Signed-Rank Test). **(C)** Flow plots for both the first (top) and second (bottom) convalescent visit of individuals where robust cTfh responses (>5%) developed. Unstimulated negative control shown for each. SARS-CoV-2 protein to which response is directed is listed next to the PTID in parentheses. Positive SARS-CoV-2-specific responses are indicated by gate frequencies in red.

To investigate the kinetics of these cTfh responses, we assessed T-cell responses in each of the convalescent individuals at a second, later visit, a median of 38 days post-symptom onset (range: 26–59 days). These first two visits were separated by a median of 14 days (range of 5–27 days). cTfh response frequencies detected by AIM increased from the first to second convalescent visit, where the overall cTfh responder rate went from 23% (6/26) to 42% (11/26). This increase in responses over time was most obviously observed towards the M protein where the cTfh response rate more than doubled from 12% (3/26) to 35% (9/26, **Fig 4A**). Additionally, M protein-specific CD4 T-cell and M protein-specific cTfh response magnitudes by AIM increased from the first to second visit (**Fig 4B**, p = 0.02 and p = 0.02, respectively).

At the first visit timepoint, there were no cTfh responses with a magnitude higher than 5%. At the second visit, five such SARS-CoV-2-specific cTfh responses were detected in four individuals. For these four individuals, the first visit took place a median of 13 days post-symptom onset, and the second visit took place a median of 33 days post-symptom onset. In the case of CID21, a robust M protein-specific cTfh response of 12.7% arose over just seven days. These antigen stimulations are shown for both Visit 1 and Visit 2 in **Fig 4C,** and the number of days between visits is indicated between the top and bottom panels. Of these responses, only one was detected at the first visit (CID11, S protein). These responses suggest that SARS-CoV-2-specific cTfh continue to increase over the first month following symptom onset.

Interestingly, these high magnitude cTfh responses do not correlate with antibody titers or antibody neutralization. At Visit 2, there is only a weak correlation seen between N protein-specific cTfh frequency and antibody titer (**S5A Fig**), and, of note, none of the high magnitude cTfh responses detected at Visit 2 are directed towards the N protein. There is no correlation between antigen-specific cTfh and antibody neutralization (**S5B Fig**). There is again no correlation between the frequency of total cTfh at Visit 2 and antibody titers/neutralization (**S5C Fig**). These findings suggest that in this subset of individuals who mount high magnitude antigen-specific cTfh responses at the second visit, the cTfh response may not be contributing to an effective antibody response. Many groups have established that SARS-CoV-2 infection can cause immune dysfunction [18–20], and it is possible that these high magnitude cTfh responses are a part of a dysregulated immune response.

It is also important to note that while responses increase from Visit 1 to Visit 2, particularly towards the M protein, not all responses initially detected at the first visit were observed at the second visit, as illustrated by the full CD4 T-cell and cTfh response mapping by AIM and ICS shown in **S6A–S6C Fig**. When considering responses detected at either timepoint, 22/26 (85%) of individuals mounted a SARS-CoV-2-specific CD4 T-cell response by AIM (**S6D Fig**), and CD4 responses were detected in 13/21 (62%) of individuals by ICS (**S6E Fig**). In fact, 55% (29/53) of CD4 T-cell responses and 71% (15/21) cTfh responses detected by AIM were found at only one of the two tested timepoints. Even so, the responder frequencies detected at each visit (62% at visit 1 and 65% at visit 2, by AIM) are slightly lower than what other recent studies have published, where SARS-CoV-2-specific T-cell responses were detected in 80–100% of individuals tested [5,7,8]. One reason for this is that we applied a stringent positivity criteria where responses were only considered positive when three times over background and significant by fisher’s exact (p value < 0.0001), based on optimization studies conducted by the HIV Vaccine Trials Network [49].

For example, for CD4 T cell responses by ICS, our responder frequency at the first visit was 48% (10/21), but if only determined by three times over background, the CD4 responder rate is 76% (16/21). Our positive response cutoff likely decreases our false positive rate but may also contribute to the discrepancy between our data and previously published studies.

### SARS-CoV-2-specific cTfh response magnitude decreases in late convalescence

**Fig 5:**
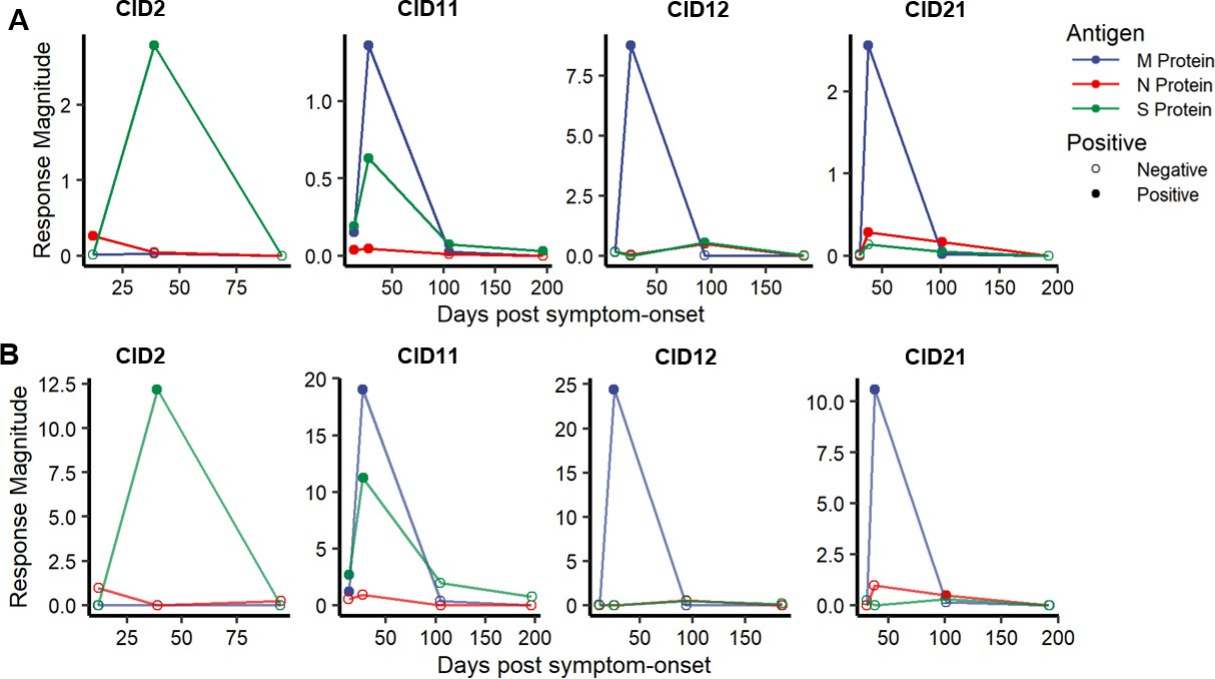
SARS-CoV-2-specific CD4 and cTfh responses decrease in magnitude in late convalescence. Frequency of AIM positive CD4 T cells **(A)** and AIM positive cTfh cells **(B)** over longitudinal convalescent visits for CID2, CID11, CID12, and CID21.

To better understand the kinetics of the robust cTfh responses detected at Visit 2, we mapped responses by AIM in these four individuals at a 3^rd^ timepoint, a median of 98 days post-symptom onset (range of 94–105). For three of these individuals, we had access to a fourth timepoint, a median of 192 days after symptoms began. Overall, we observed a contraction of CD4 and cTfh responses between Visit 2 and Visit 3 but still detected several CD4 T-cell responses and one cTfh response at Visit 3 (**Fig 5A and 5B,** flow plots shown in **S7 Fig**). While no cTfh responses were detected beyond the 3^rd^ visit, in CID11, an S protein-specific CD4 T-cell response was detected at Visit 4, 196 days after symptom-onset. The overall trend of the CD4 T-cell and cTfh responses is consistent with the contraction of a primary T-cell response induced by acute infection, but a few long-lived CD4 T-cell responses are still detected late in convalescence.

CD4 T-cell responses in CID11 and CID21 were detected longitudinally: in CID11, an S protein-specific response was detected at Visit 2, 3, and 4, and in CID21, M and N protein responses were detected at both Visit 2 and 3 (**S7A and S7B Fig**). Surprisingly, we also detected new N and S protein-specific CD4 T-cell responses in CID12 at Visit 3, 94 days after symptom onset, which were not detected at the first two visits. We also observed a new S protein-specific CD4 T-cell response in CID21, 101 days post-symptom onset (**S7A Fig**). Only one cTfh response was detected at Visit 3; an N protein-specific response in CID21 that was not positive over background at Visit 2 (**S7C Fig**). These data suggest that CD4 T-cell and cTfh responses can be detected in circulation several months after symptom onset, which could be due to a delayed T-cell response phenotype or might be a result of persistent antigen presentation long after initial SARS-CoV-2 infection [50].

## Discussion

In this study, we longitudinally examined the CD4 T-cell responses targeting the major SARS-CoV-2 structural proteins, M, N, and S, in 26 convalescent individuals by measuring the expression of activation markers and the production of effector cytokines. We found that at the first convalescent visit, antigen-specific cTfh responses could be detected against all three proteins and that the frequency of antigen-specific cTfh in these individuals correlated with nAb, albeit to varying degrees. We also found that cTfh responses increase over time in convalescence, particularly M protein-specific ones, that truly robust cTfh responses (>5% frequency) were only detected at a second visit, and that these responses eventually undergo contraction late in convalescence.

The relative weakness of the correlation between N protein-specific cTfh frequency and antibody neutralization compared to the M and S proteins may relate back to the structure of SARS-CoV-2. Both the spike and membrane proteins have portions that are located exteriorly, while the nucleocapsid protein is found exclusively internally. Collectively, these data suggest that cTfh responses induced against different SARS-CoV-2 proteins may not be equally effective in aiding B cells and bolsters the foundation for vaccine strategies recently approved by the FDA under emergency use authorization and those currently in testing which only include the Spike protein. In fact, many of these vaccines have reported levels of antibodies similar to those seen in natural SARS-CoV-2 infection and mild disease, which may be a result of focusing the cTfh response on the S protein [12,14]. However, as prior HIV studies have shown, CD4 T cells across different protein specificities may contribute to nAb induction [45,46]. Future studies should work to ascertain the level to which M and N protein-specific cTfh responses contribute to the formation of neutralizing antibodies. It is possible that cTfh responses across different protein specificities all play a synergistic role in the development of nAb.

Interestingly, the robust cTfh responses detected at the 2^nd^ timepoint were only found against the M and S proteins, not the N protein, and the correlation between cTfh response magnitude and antibody neutralization no longer existed at this later timepoint. Many groups have described significant T-cell dysfunction in acute SARS-CoV-2 infection [18,19,51], and our group has recently illustrated that this dysfunction is sustained during convalescence, even in nonhospitalized individuals [20]. It is possible that the robust cTfh responses detected in this subset of individuals is evidence of a dysregulated immune response and that these cTfh do not contribute to nAb formation. Future studies would ideally delve deeper by examining additional relevant cytokines, like IL4, IL13, and IL21, and combine activation marker and cytokine staining to allow for comprehensive functional analysis of the CD4 T cells arising later in convalescence.

A more comprehensive assessment is also important as some SARS-CoV-2-specific CD4 T cells may not express the traditional surface markers or produce typical cytokines as a result of this immune dysfunction.

Meanwhile, the observed increase in cTfh responses over time suggests that cTfh response formation may also be delayed in SARS-CoV-2 infection. A study of influenza vaccination showed that cTfh responses peaked seven days after vaccine administration [31]; meanwhile, a longitudinal study of cTfh in dengue virus infection found that the frequency of antigen-specific cTfh decreased from the time of acute infection [28]. In mouse models of influenza infection, cTfh responses peak in the first week after infection and have undergone contraction well within the first month of infection [52,53]. In comparison with these studies, it appears that cTfh response formation in SARS-CoV-2 infection continues farther into convalescence than expected—the second visit for all individuals assessed in this study occurred a median of 38 days following symptom onset. Although we did not observe cTfh responses in late convalescence, other groups have detected longitudinal cTfh responses up to 6 months after initial symptom onset [43,44]. These studies classified cTfh cells as CXCR5^+^ (no PD1) and investigated activation markers other than Ox40 and PDL1. Taken together, future investigation into longitudinal cTfh responses is warranted. A delay in cTfh response formation could be due to the T-cell dysfunction that occurs in SARS-CoV-2 infection. These high magnitude cTfh responses could also be the result of persistent antigen exposure, as several groups have reported prolonged detection of SARS-CoV-2 by PCR [50,54]. Prolonged cTfh responses could also go hand in hand with recent reports of B cell and antibody response evolution over time in natural infection [55]. However, our understanding of the natural kinetics of cTfh responses in viral infections is incomplete, and future studies should examine the frequency and magnitude of these responses over time in SARS-CoV-2 infection, but also in influenza and other common viral infections.

These data further our understanding of CD4 T-cell responses, particularly cTfh responses, against SARS-CoV-2. Our study directly measures SARS-CoV-2-specific cTfh responses to three major structural proteins, M, N, and S. We clearly demonstrate that SARS-CoV-2-specific cTfh responses that arise early in convalescence strongly correlate with antibody neutralization and that S protein-specific responses most closely relate to antibody neutralization. However, we also show that cTfh responses against other SARS-CoV-2 proteins early in convalescence correlate with antibody neutralization, indicating a possible role for intrastructural help. Finally, in measuring these responses over time, we observe the emergence of several high magnitude responses more than a month following symptom onset, suggesting that cTfh response formation may be delayed in SARS-CoV-2 infection. These responses undergo contraction within the following two months and do not correlate with antibody neutralization, suggesting they may represent immune dysregulation.

## Methods and materials

### Ethics statement

All patients included in this study were adults and recruited from the University of Alabama at Birmingham (UAB) HIV care clinic, also known as the 1917 clinic, after obtaining written, informed consent and approval from the Institutional Review Board (IRB-160125005) at UAB.

### Patient samples

Cryopreserved PBMC samples for T-cell assays and plasma samples for antibody assays were acquired through the UAB COVID Enterprise Biorepository. All samples were obtained with patient consent under the appropriate IRB guidelines. Patient demographic information is shown in **Table 1**. Paired Visit 1 and Visit 2 PBMC samples from 26 individuals who had recovered from COVID-19 were assessed in this study. 4 of these individuals were tested at additional later timepoints. CID2, CID11, CID12, and CID21 were tested at Visit 3, and CID11, CID12, and CID21 were tested at Visit 4. Clinical data from these individuals were retrieved from the Enterprise Biorepository REDCap database [56]. All tested individuals were symptomatic, but none were hospitalized during the course of their illness. Symptom severity was quantified using a self-reported severity score on a scale of 1 to 3, where 1 represented no interference in daily life, 2 a moderate impact on daily life, and 3 a significant decrease in quality of life due to symptoms. A majority of individuals reported moderate severity (62%, 16/26) and a minority reported mild severity of symptoms (38%, 10/26). None reported severe symptoms. Additionally, all but two had a positive SARS-CoV-2 PCR by nasopharyngeal swab. The two individuals who did not have a PCR test completed had a known COVID contact, were symptomatic, and had detectable T-cell responses. PBMCs from 10 healthy donors (all collected prior to the COVID-19 pandemic) were assessed for T-cell responses in parallel.

### Peptide pools

Overlapping peptides spanning the SARS-CoV-2 M, N, and S proteins (NCBI reference number MN985325.1) were designed as 20mers overlapping by 10 amino acids which has previously been shown to effectively detect CD4 T-cell responses [57,58]. Peptides were synthesized by New England Peptide in a 96-well plate format and stored at -70°C after reconstitution in DMSO.

### Flow cytometry

For activation-induced marker (AIM) staining, cells were thawed and stimulated with SARS-CoV-2 peptide pools for each of the M, N, and S proteins. An unstimulated, negative control and an SEB stimulated, positive control were included for each sample and were incubated and stained in parallel with experimental conditions. The unstimulated cells were cultured with 1% DMSO to best account for background activation levels. Co-stimulatory anti-CD28 and anti-CD49d were added (BD Pharmingen). After an 18 hour incubation at 37°C, cells were washed with FACS wash (2% FBS in PBS), stained with CCR7-PercpCy5.5 at 37°C for 20 min, washed, and then stained with the following antibodies: CD4-Pe610, CD3-A780, CD8-FITC, CD14-A700, CD19-A700, Ox40-PeCy7, PDL1-PE, CXCR5-BV421, PD1-BV785, CD45RABV510, CD137-BV650, CD69-BUV737, and Dead cell dye-UV. Cells were then washed and fixed in 2% formaldehyde. Events were collected on a BD FACSymphony A3 within 24 hours and analyzed using FlowJo software (v10). PD1 gating was set by a fluorescence minus one control (FMO).

Intracellular staining (ICS) experiments were set up in parallel with the AIM staining experiments for 21 of the 26 individuals and performed similarly, with a few notable exceptions.

CD107a-FITC was added with the co-stimulatory antibody mix; Monensin and Brefeldin A (BD Bioscience) were added after 1 hour. Cells were incubated for 12 hours in total, instead of 18. Staining was conducted in three steps: 1) Surface marker staining for 30 min at 4°C with Dead cell dye-UV, CD3-A780, CD4-BV785, CD8-V500, CD14-PercpCy5.5, and CD19-PercpCy5.5. 2) Permeabilization with CytoFix/CytoPerm solution (BD Biosciences) for 20min at 4°C. 3) Intracellular staining for 30 min at 4°C with IFNγ-A700, TNFα-PeCy7, and CD154-APC. CD154 was plotted against IFNγ. Additional details regarding the antibodies used in both the AIM and ICS assays can be found in **S1 Table**, and the gating strategies for AIM and ICS in an unstimulated, negative control are shown in **S1 Fig**. For both AIM and ICS, positive responses were determined as those at least three times higher than the unstimulated control and significantly higher than unstimulated control by Fisher’s Exact (p value < 0.0001).

### Antibody assays

Plasma samples from the first and second time point were tested for SARS-CoV-2-specific antibodies. The Abbott Architect assay was used to detect immunoglobin G (IgG) reactivity to the SARS-CoV-2 nucleocapsid protein [59]. The IgG quantity is reported as a calculated index specimen/calibrator ratio, and values over 1.4 were considered positive for N protein IgG. Manufacturer-reported specificity of this assay is 99.6% (99.1%-99.9%).

Antibody neutralization assays were conducted as previously described [60]. Briefly, the SARS-CoV-2 Spike (G614 variant, with a 19 amino acid cytoplasmic tail deletion) was pseudotyped onto an HIV-1 nanoluciferase reporter backbone by co-transfection in HEK 293T cells. Pseudovirus was incubated with five-fold serial dilutions of patient plasma and then used to infect 1.5x10^4^ 293T clone 22 cells expressing ACE2. Two days post-infection, cells were washed with PBS, lysed, and nanoluciferase activity was determined according to manufacturer’s instructions (Nano-Glo Luciferase Assay System). Luciferase activity in wells with virus and no patient plasma were set to 100%, and the dilution of plasma at which luminescence was reduced to 50% (ID50) was calculated. Abbott IgG titer was not conducted for the second timepoint for two individuals, CID2 and CID13. Otherwise, antibody measurements were collected for all 26 individuals.

### Statistical analysis

Comparisons between paired visit 1 and visit 2 magnitudes were conducted by Wilcoxon signed-rank tests. Comparisons between responder frequency at visit 1 and visit 2 were conducted by Fisher’s exact tests. All correlations were determined by Spearman Rank tests, with the exception of S2A Fig, where multiple measurements were plotted for each individual (across the three proteins) and therefore a generalized linear mixed effect model accounting for multiple measurements per individual was employed. In Figs 3 and S4, axes were transformed using log10(x+1) to allow for visualization of zeros, and correlations were determined with untransformed data.

## Supporting information

S1 Fig Flow cytometry gating strategies. **(A)** Gating strategy for CD4 T cell and cTfh by activation-induced marker (AIM). **(B)** Gating strategy for CD4 T cell staining by intracellular cytokine staining.
(TIF)

S2 Fig SARS-CoV-2-reactive CD4 T cells are infrequently detected in COVID negative individuals. Representative examples of CD4 T-cell responses detected in COVID negative individuals by upregulation of activation-induced markers **(A)** and by intracellular cytokine staining **(B)** upon stimulation by SARS-CoV-2 N protein peptide pool. Responder frequency of CD4 responses to any SARS-CoV-2 protein and to the M, N, and S proteins individually by AIM **(C)** and ICS **(D)**. Positive SARS-CoV-2-specific responses are indicated by gate frequencies in red.
(TIF)

S3 Fig Upregulation of activation markers detected a broader range of SARS-CoV-2-specific CD4 T-cell responses. Correlation between response magnitude by AIM versus response magnitude by ICS. Statistics determined by mixed effect model accounting for multiple protein stimulations per individual, and correlation represented by linear regression line. Data transformed by log10(x+1) to allow for visualization of 0s.
(TIF)

S4 Fig Nine SARS-CoV-2-specific cTfh responses were detected at the first convalescent visit. Each row shows responses from a different individual. From right to left, unstimulated, media control; M protein, N protein, S protein stimulations; and positive, SEB-stimulated control. Positive SARS-CoV-2-specific responses are indicated by gate frequencies in red.
(TIF)

S5 Fig cTfh response frequency does not correlate with antibody neutralization at Visit 2. **(A)** Correlations between N protein IgG titers and cTfh frequencies towards the M, N, and S proteins. **(B)** Correlations between antibody neutralization (ID50, dilution of plasma at which luminescence was reduced to 50%) and cTfh frequencies. **(C)** Correlations between the total cTfh frequency and antibody titer and neutralization. (All correlations represented by a linear regression line. Y axis in A-B and both axes in C are transformed by log10(x+1) to allow for visualization of 0s. Statistics determined by a Spearman Correlation test. Points are colored for each individual.)
(TIF)

S6 Fig Summary of all responses detected across the first two convalescent visits. **(A-C)** Response summary for CD4 T cells by activation-induced marker staining, for cTfh by activation-induced marker staining, and for CD4 T cells by intracellular cytokine staining, respectively. Blue-filled cells indicate a positive response; white cells indicate a negative response. **(D)** Responder frequency by AIM across Visit 1 and Visit 2 (positive at either visit) overall and to each protein. **(E)** Responder frequency by ICS across the first two visits (positive at either visit).
(TIF)

S7 Fig CD4 T-cell and cTfh responses can be detected late in convalescence. Each row shows CD4 T-cell **(A)** and cTfh **(B)** responses from a different individual. From right to left, unstimulated, media control; M protein, N protein, S protein stimulations; and positive, stimulated control. Positive SARS-CoV-2-specific responses are indicated by gate frequencies in red.
(TIF)

S1 Table Flow cytometry panels. Details of antibodies used for activation-induced marker flow cytometry and intracellular staining flow cytometry.
(TIF)
