# Supplementary figures and images for: SARS-CoV-2-specific circulating T follicular helper cells correlate with neutralizing antibodies and increase during early convalescence
Source: PLoS Pathog. 2021 Jul 16;17(7):e1009761. doi: 10.1371/journal.ppat.1009761 (PMC8318272; doi:10.1371/journal.ppat.1009761)

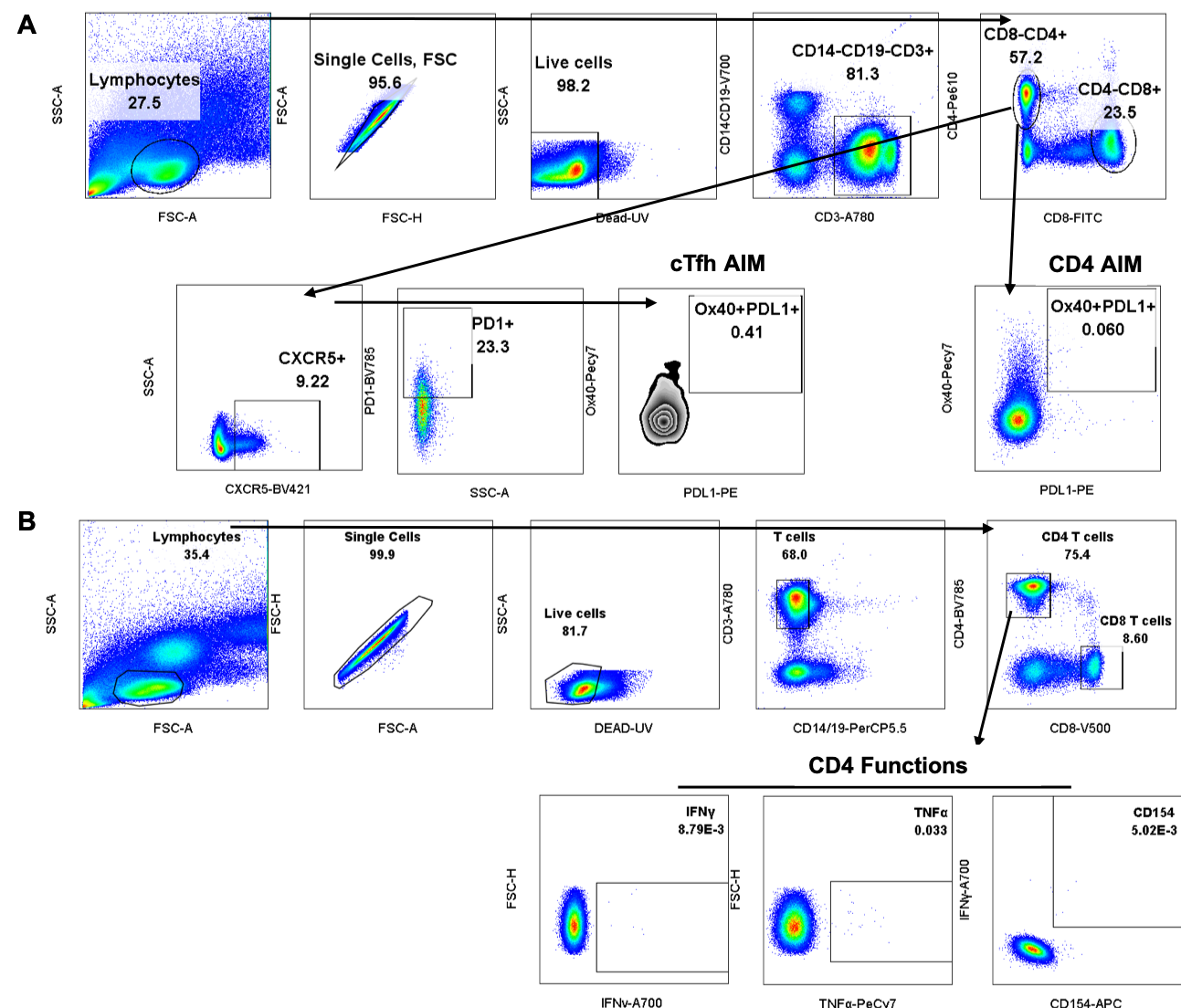

Supplement: S1 Fig — (A) Gating strategy for CD4 T cell and cTfh by activation-induced marker (AIM). (B) Gating strategy for CD4 T cell staining by intracellular cytokine staining. (TIF) [file ppat.1009761.s001.tif]

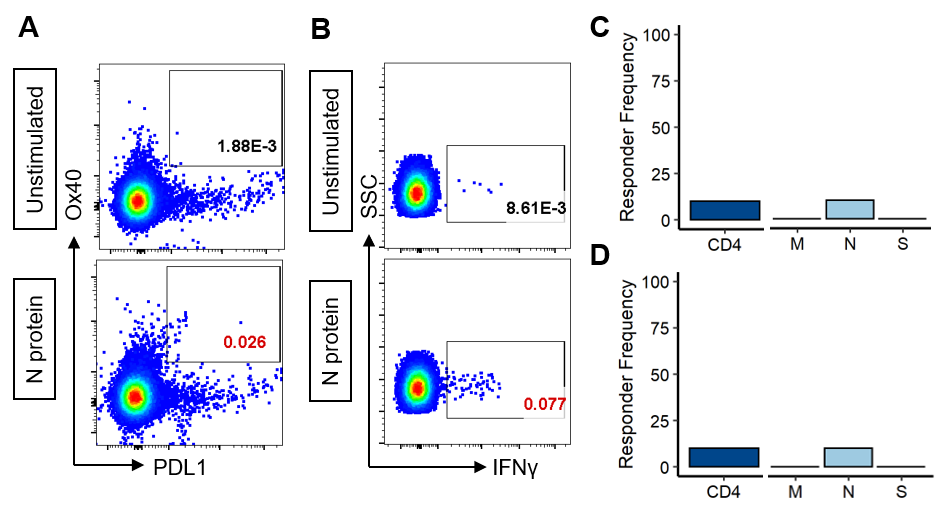

Supplement: S2 Fig — Representative examples of CD4 T-cell responses detected in COVID negative individuals by upregulation of activation-induced markers (A) and by intracellular cytokine staining (B) upon stimulation by SARS-CoV-2 N protein peptide pool. Responder frequency of CD4 responses to any SARS-CoV-2 protein and to the M, N, and S proteins individually by AIM (C) and ICS (D). Positive SARS-CoV-2-specific responses are indicated by gate frequencies in red. (TIF) [file ppat.1009761.s002.tif]

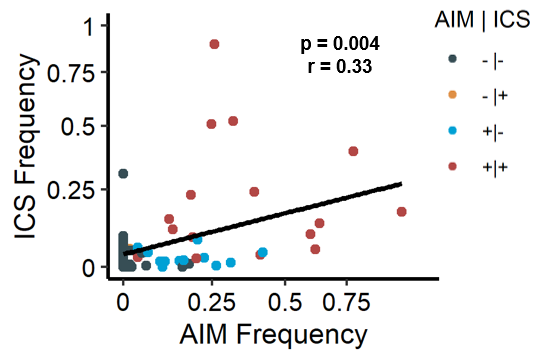

Supplement: S3 Fig — Correlation between response magnitude by AIM versus response magnitude by ICS. Statistics determined by mixed effect model accounting for multiple protein stimulations per individual, and correlation represented by linear regression line. Data transformed by log10(x+1) to allow for visualization of 0s. (TIF) [file ppat.1009761.s003.tif]

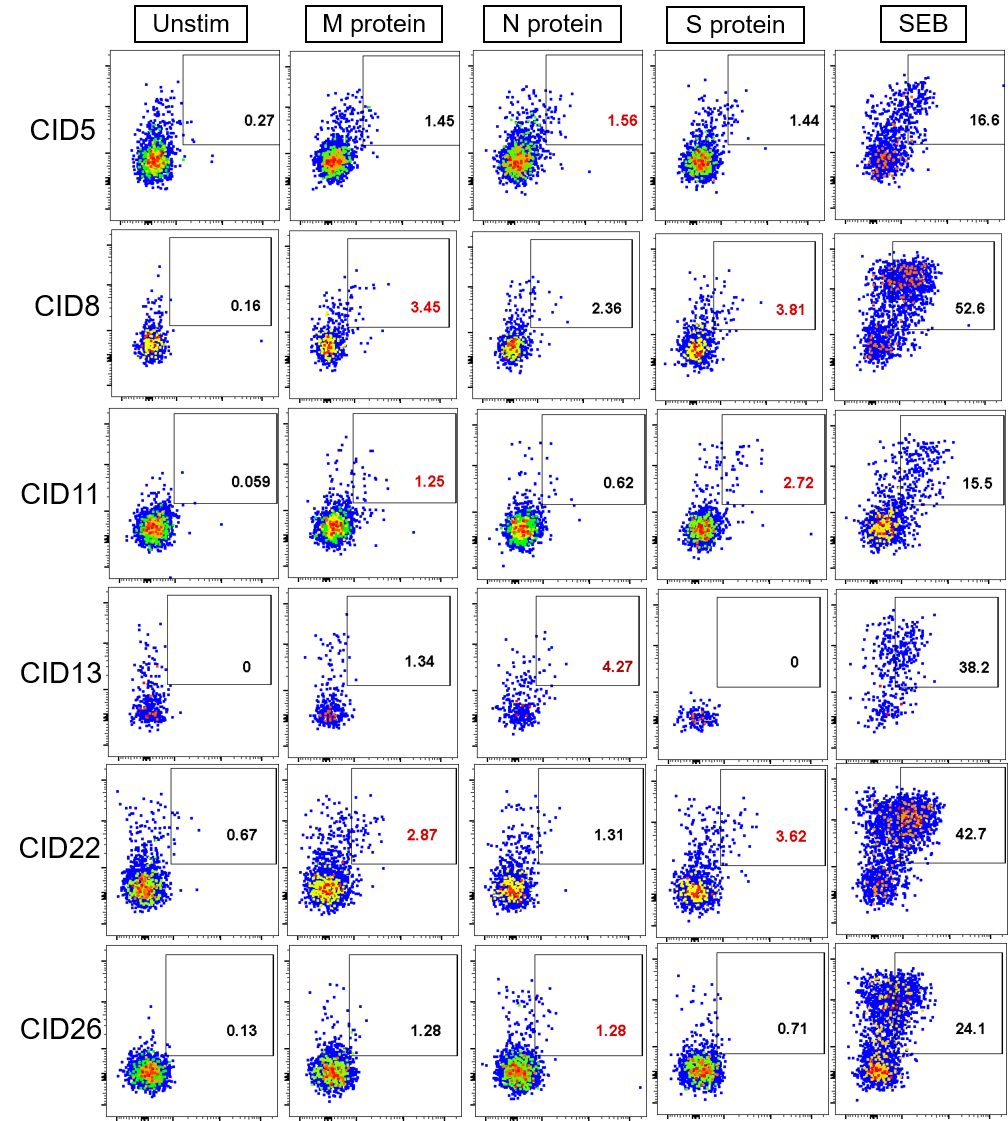

Supplement: S4 Fig — Each row shows responses from a different individual. From right to left, unstimulated, media control; M protein, N protein, S protein stimulations; and positive, SEB-stimulated control. Positive SARS-CoV-2-specific responses are indicated by gate frequencies in red. (TIF) [file ppat.1009761.s004.tif]

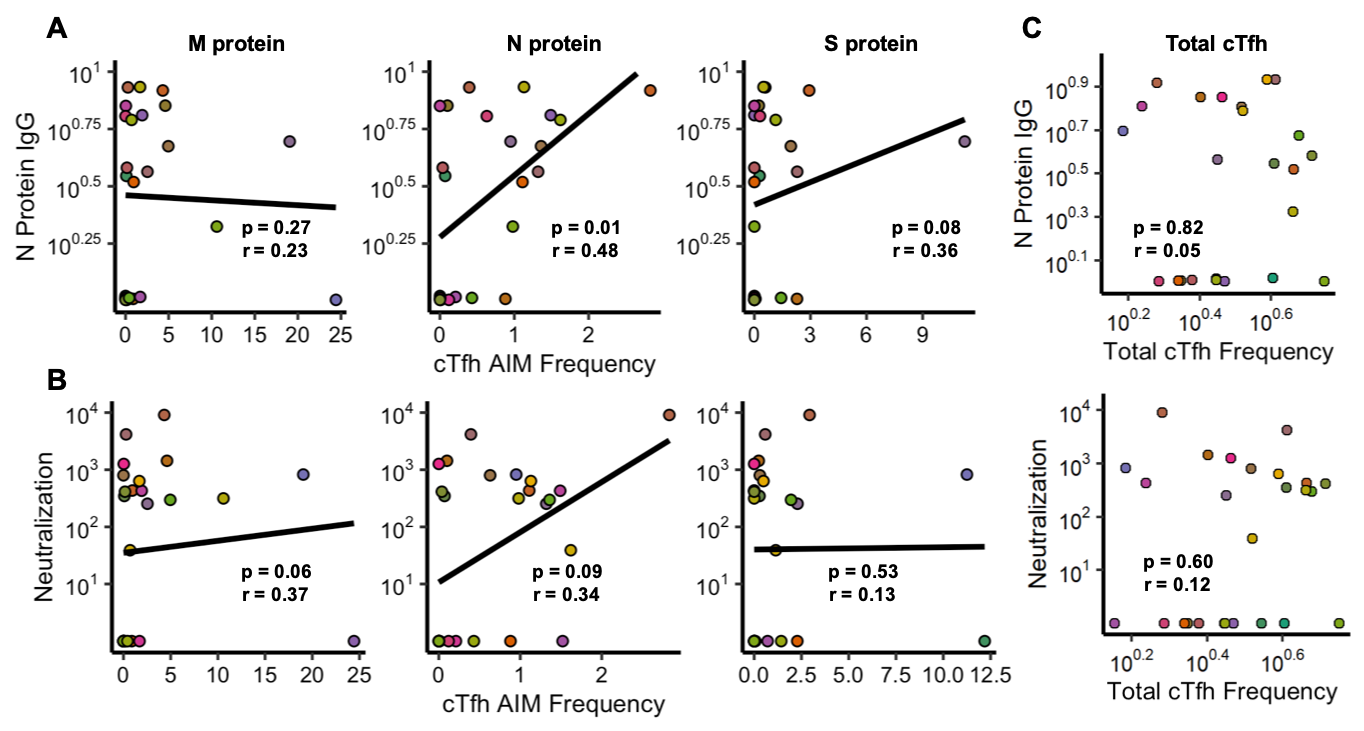

Supplement: S5 Fig — (A) Correlations between N protein IgG titers and cTfh frequencies towards the M, N, and S proteins. (B) Correlations between antibody neutralization (ID50, dilution of plasma at which luminescence was reduced to 50%) and cTfh frequencies. (C) Correlations between the total cTfh frequency and antibody titer and neutralization. (All correlations represented by a linear regression line. Y axis in A-B and both axes in C are transformed by log10(x+1) to allow for visualization of 0s. Statistics determined by a Spearman Correlation test. Points are colored for each individual.) (TIF) [file ppat.1009761.s005.tif]

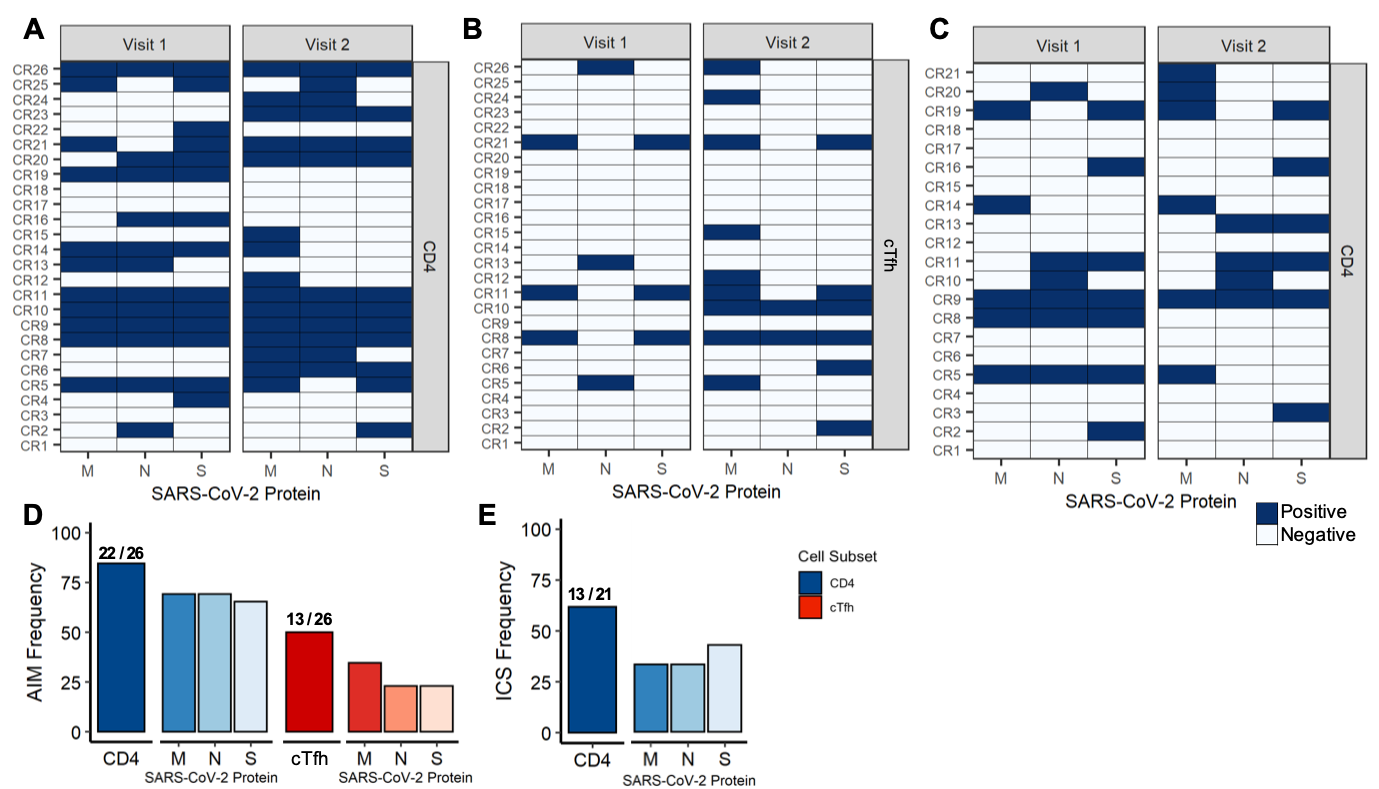

Supplement: S6 Fig — (A-C) Response summary for CD4 T cells by activation-induced marker staining, for cTfh by activation-induced marker staining, and for CD4 T cells by intracellular cytokine staining, respectively. Blue-filled cells indicate a positive response; white cells indicate a negative response. (D) Responder frequency by AIM across Visit 1 and Visit 2 (positive at either visit) overall and to each protein. (E) Responder frequency by ICS across the first two visits (positive at either visit). (TIF) [file ppat.1009761.s006.tif]

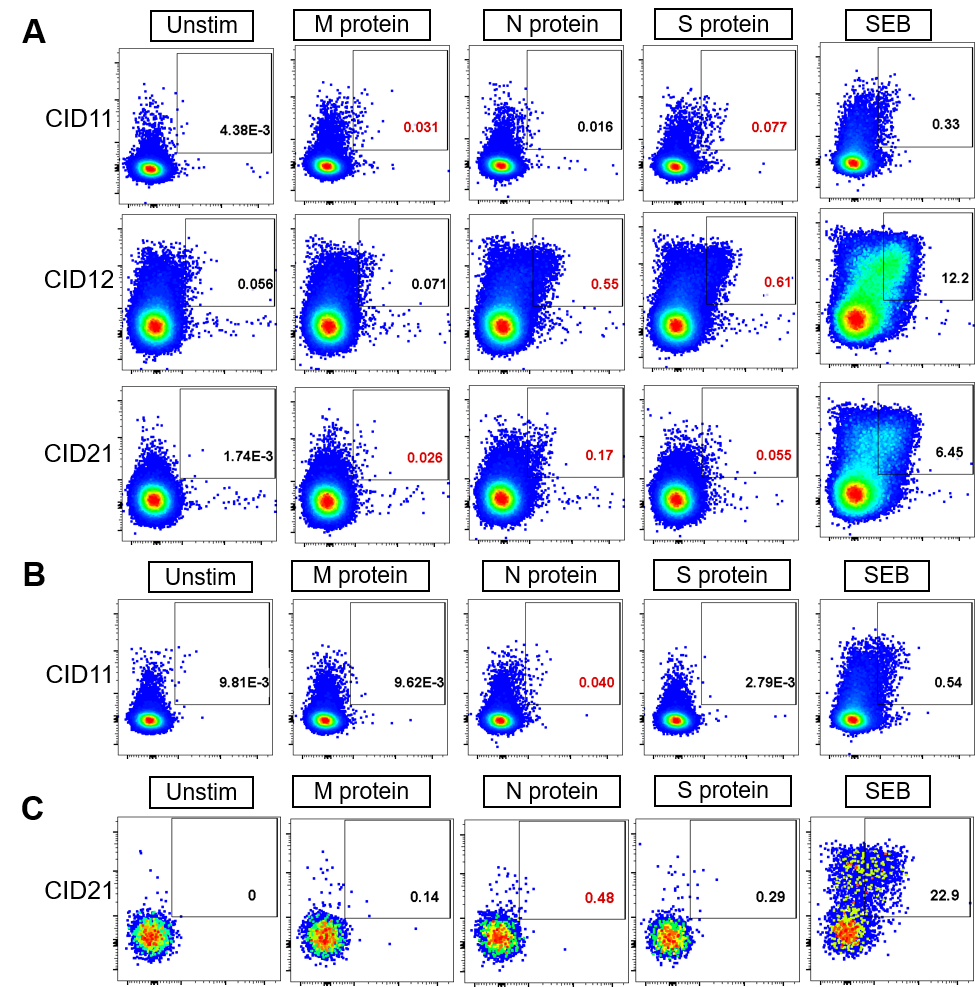

Supplement: S7 Fig — Each row shows CD4 T-cell (A) and cTfh (B) responses from a different individual. From right to left, unstimulated, media control; M protein, N protein, S protein stimulations; and positive, stimulated control. Positive SARS-CoV-2-specific responses are indicated by gate frequencies in red. (TIF) [file ppat.1009761.s007.tif]

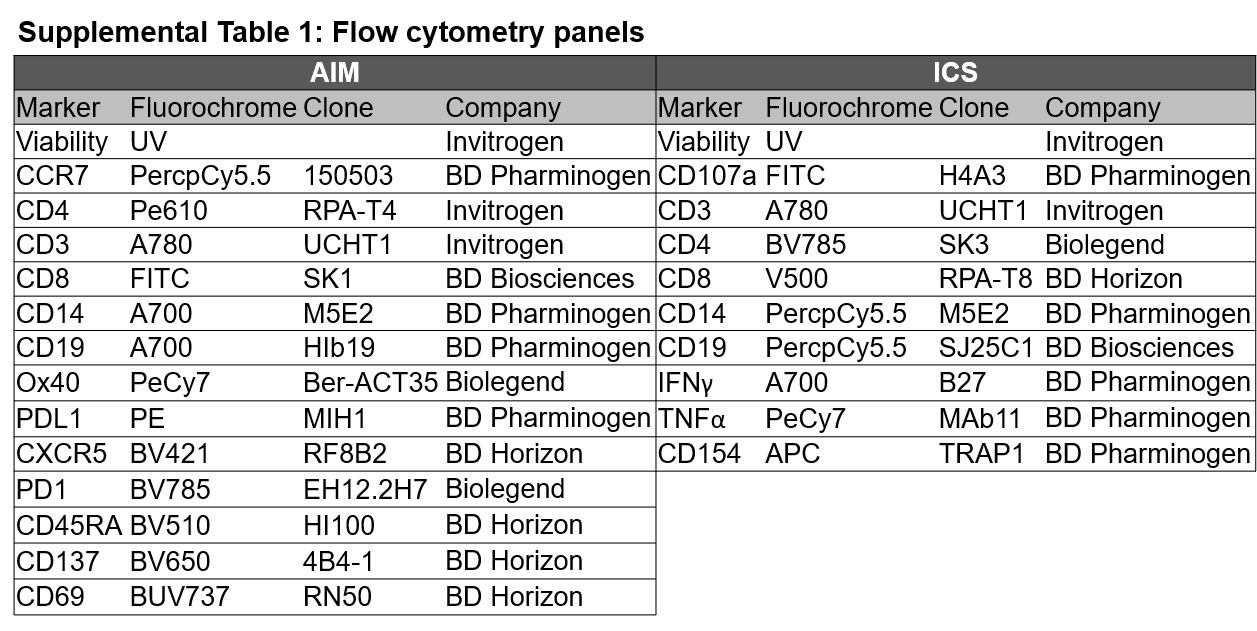

Supplement: S1 Table — Details of antibodies used for activation-induced marker flow cytometry and intracellular staining flow cytometry. (TIF) [file ppat.1009761.s008.tif]
