# Supplementary material for: SARS-CoV-2-specific circulating T follicular helper cells correlate with neutralizing antibodies and increase during early convalescence
Source: PLoS Pathog. 2021 Jul 16;17(7):e1009761. doi: 10.1371/journal.ppat.1009761 (PMC8318272; doi:10.1371/journal.ppat.1009761)
Supplement: Attachment — Submitted filename: Initial_response_to_reviewers.pdf [file ppat.1009761.s009.pdf]

Reviewer's Responses to Questions

Part I - Summary

Please use this section to discuss strengths/weaknesses of study, novelty/significance, general execution and scholarship.

Reviewer #1: The authors measure antigen-specific pTfh responses in 21 COVID-19 individuals at two convalescent timepoints using the OX40/PDL1 AIMS assay and the IFN-gamma/CD154 ICS assay for comparison. Overlapping peptide pools to S, N and M were used for 18 hrs of stimulation. They have classified pTfh cells as CD4+CXCR5+PD-1+ and compared those responses with the overall CD4+ T cell subset. A similar study has previously been performed by Juno et al. (Nature Medicine), as cited by the authors, where they measured pTfh responses to Spike and RBD proteins/peptides by gating on memory CD4+CXCR5+CD45RA- pTfh cells before further sub-setting based on CXCR3 and CCR6. It should be noted that PD-1 was added into their panel but was not shown in their publication and hence this current study is not entirely novel with the "PD-1" addition. The use of overlapping S, N and M peptides has also been shown to elicit robust CD4+ T cell responses in convalescent individuals using expansion and ICS techniques in a recent study by Habel et al. (PNAS) and should also be cited, although they did not look at pTfh responses.

We have now cited the research done by Habel et al in the introduction (**Ref 8**) and have clarified our discussion Juno et al, stating that PD1 was included in their panel but not used for defining pTfh or reported in their results.

The authors claim that pTfh responses increase during convalescence in the title, however the evidence is not strong enough to support this statement. While the trend looks to increase, it does not reach significance, and as the authors noted in the discussion and Fig S4, some responses were only detectable at visit 1. The authors need to tone down their conclusions and remove from the title.

We agree that our original data was not strong enough to support our title stating that pTfh responses increase during convalescence. To bolster our data, we tested an additional 5 individuals at visits 1 and 2 to measure CD4 and pTfh AIM responses to the M, N, and S proteins of SARS-CoV-2. By increasing our sample size to 26, we actually observed a significant increase in the magnitude of response from Visit 1 to Visit 2 for both the detected CD4 T-cell responses and pTfh responses to the SARS-CoV-2 M protein ($p = 0.02$ each, **Fig 4B**). Based on these data, we believe the title supports our main conclusions.

Peripheral Tfh responses peaking at day 7 following influenza vaccination generally arises from a boost in memory responses, whereas the pTfh response observed in convalescent COVID-19 individuals is following a primary SARS-CoV2 infection, and so it is not surprising that these pTfh responses take longer to develop, and hence are later detected in the peripheral blood, in addition to the T cell dysfunction as the authors have described and should also be considered in the discussion.

We completely agree that it is difficult to place these data in context of naturally induced pTfh in other viral infections due to a lack of literature on natural infection-induced pTfh. To address this point, we have expanded our discussion by including some data on influenza infection in mouse models in **Lines 326-333**.

Overall, the study does add to the current knowledge on pTfh cells and their correlations with the antibody response to COVID-19 during the first 2 months after infection.

Reviewer #2: This is an interesting look at the reactivity of CD4 T cells to several SARS-CoV-2 proteins in COVID convalescent subjects. The preliminary report is somewhat descriptive and it is difficult to know what new insight is gained from this study at this early stage of the work. Also, some of the methods were inadequately described and with some controls confusing.

Much of the data come from the use of the AIM assay, and these methods need rigorous controls. For example, it is not clear if the "unstimulated" control sample has been cultured with control peptides, media/solvents alone or simply analyzed directly after thaw. This is particularly an issue with the supplementary data shown in Supplementary Figure 3, where there is significant disparity between the numbers of SARS reactive cells detected by cytokine production and by the AIM assay. With two cytokines measured, it is difficult to know if many of these low frequency cells are false positives by the AIM assay or simply cells that do not produce these two cytokines. The data provided in Supplementary Figure 4 as positive or negative emphasizes the ability to extract generalizable in the specificity of the CD4 T cell responses and the reliability of the thresholds used to assign a positive or negative response.

We have further clarified our AIM methodology in the methods section (**Lines 383-395**). Specifically, we noted that unstimulated and positive controls were incubated and stained in parallel with experimental conditions. The unstimulated cells were cultured with 1% DMSO to best account for background activation levels.

In terms of possibility of false positives by the AIM assay, we believe our positivity criteria is highly stringent and that we are more likely to miss low magnitude responses than to result in false positives. Our positive criteria is at least 3 times background and significantly higher than background by fisher's exact p value < 0.0001 (added in **Lines 402-405**). We agree that it will be important for future studies to delve into better understanding the functionality of these CD4 T cells detected by AIM and not by ICS.

Also, it is not clear how the gates were constructed for the examples shown in Figure 2 (whether by the negative "unstimulated" control or via FMO). It is also not clear why the examples of the AIM assays shown in Figure 2 as examples, chose 3 different subjects for the three different antigens. It would have been more useful to be less selective and provide several examples of these reactivities with each of the subjects who were scored as positive. Overall, the quantification of antigen reactive cells by the AIM assay was not compelling and thus the secondary data from this quantification, such as relationships to antibody responses, is difficult to interpret rigorously.

Since our group does multi-color flow cytometry, we routinely use FMO to determine appropriate gating of populations especially for markers where discrete immune subsets are not easily discernible. We have now added more details regarding the methodology used in the AIM assay, clarifying that PD1 gating was determined by FMO and positive response were determined relative to background (unstimulated control) in each individual (**Lines 381-383 and 390-391**). To provide more examples of reactivities (negative/positive, based on our stringent criteria), we have added the flow data from all individuals with a SARS-CoV-2-specific pTfh response in **S4 Fig**.

A second difficulty in this paper is the choice of antigens for studies of CD4 T cell reactivity. The authors do not justify their choice but it is curious that the M protein is only about 1/5 the length as spike (and presumably possesses a proportionate of CD4 T cell epitopes) and yet recruits approximately as many Tfh and total CD4 (Figure 4). The most likely explanation for this is that because of the significant sequence identity between among the M protein from seasonal HCoV and that from SARS-CoV-2, the authors are seeing recall and selective expansion of pre-existing memory cells. They do not comment on this or on whether they have any evidence if the cross-reactive CD4 T cells have typical helper function.

For this study, we used pools of peptides spanning 20 amino acids and overlapping by 10 amino acids. This overlapping peptide pool strategy is quite commonly used to detect CD4 T-cell responses (Tobery et al 2001 *J Immunol Methods*, Draenert et al 2003 *J Immunol Methods*). In fact, several of the SARS-CoV-2 publications to date have used peptide pools to stimulate antigen-specific T cells (Braun 2020 *Nature*, Le Bert 2020 *Nature,* Grifoni 2020 *Cell*).

In examining sequence homology between SARS-CoV-2 and seasonal coronaviruses, we do not observe a higher degree of similarity of the M protein compared to the S protein (**Figure R1**). Additionally, from studies in HIV, we know that the length of a protein does not necessarily correlate with immunogenicity: the Nef protein is only 200-215 amino acids long but is the most commonly targeted protein in HIV-1 infection by T-cell responses (Wang et al 2005 *JVI*, Mlotshwa et al 2010 *JVI*).

Additionally, we find it reassuring that we rarely see SARS-CoV-2-specific CD4 T-cell responses in the COVID negative individuals and that we do not detect any SARS-CoV-2-specific pTfh responses in the COVID negative individuals. Though we are unable to distinguish whether these responses are an expansion of cross-reactive responses or new responses, but because they are preferentially observed in SARS-CoV-2 positive individuals, these responses are likely induced by recent SARS-CoV-2 infection.

Figure R1: Sequence Homology of SARS-CoV-2 M and S Proteins to Seasonal Coronavirus Strains. Sequence homology determined as proportion of amino acids conserved scanning 10mers aligned between each seasonal coronavirus strain and SARS-CoV-2. Homology above dotted line at 0.70 indicates an increased possibility of cross-recognition based on past studies (Boppana et al 2019 *JID*).

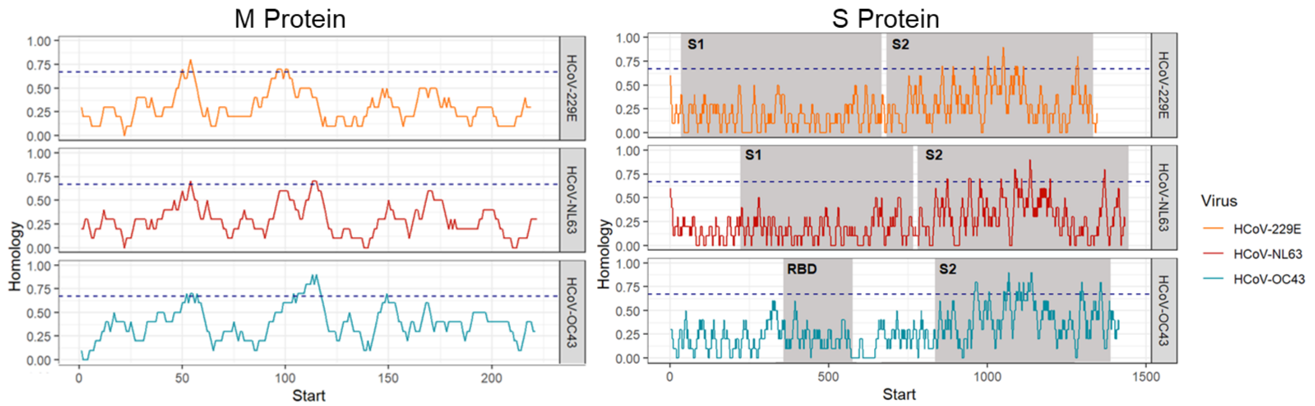

Unfortunately, our data cannot distinguish between de novo responses and cross-reactive ones. We agree that this is an interesting question, and we think that the kinetics of these responses align more closely with *de novo* responses; we would expect cross-reactive responses to arise earlier in infection. However, this specific question is outside the scope of our paper but we look

forward to future studies that look more closely at separating out cross-reactive versus *de novo* responses.

The kinetics features of the Tfh are interesting but the authors do not provide any comparator for this or discuss if this kinetic feature is similar to what has been observed in response to a different respiratory pathogen such as influenza, that typically resolves normally. The authors compare the kinetics to influenza vaccination, which will be a synchronous response with a finite antigen load and to dengue infection, where the “late” time point was a year post peak. Thus it is not clear whether this slowly evolving Tfh response is atypical. If the authors have complementary data on post influenza Tfh, a reasonable comparator which show a different pattern of kinetics, inclusion of these data would add to the impact of their studies. The somewhat unpredictable agreement among the assays shown in Supplementary Figure 4 across the two time points, emphasizes the challenges in interpretation of these data.

We completely agree that a comparison between SARS-CoV-2-induced pTfh responses to those seen in influenza infection would be an incredibly informative study, but we feel that this is outside the scope of this paper. To help put our data in context of other viral infections, we have added some discussion to this point (**Lines 326-333**). Additionally, to more fully illustrate the kinetics of these pTfh responses, in the four individuals who mounted robust pTfh responses at Visit 2, we screened for response at later timepoints, > 90 days after symptoms onset. We detect SARS-CoV-2-specific CD4 T-cell responses in 3 of the 4 individuals tested and one pTfh response at Visit 3. One of these long-lived CD4 T-cell responses was detectable even at the last timepoint tested, 190 days post-symptom onset (**Figure 5**, **Supplemental Figure 7**).

Overall, these are valuable samples and the authors have provided the results of some interesting first layer analyses, but the depth of the primary experiments shown is quite limited and in many places, difficult to interpret.

Reviewer #3: This paper describes the assessment of CD4 T cell responses in convalescent COVID-19 patients who experienced moderate disease severity. A major strength of the study include the analysis of M, N, and S -specific CD4 T cell responses and specifically peripheral Tfh responses using activation induced marker (AIM) assays and cytokine secretion assays, and the correlation of these antigen specific CD4 T cell responses with the generation of neutralizing antibody responses. An additional strength of the study includes the indication of responder rates for CD4 T cell responses among the patients following recovery. The most surprising and provoking aspect of the study is that in many recovered patients, their Ag-specific pTfh cell responses continue to increase upon the second study visit (around day 36 after symptom onset), indicating a delayed kinetics of the Tfh response. Weaknesses or drawbacks of the study include the fact that former studies of Tfh responses in COVID-19 patients have described the correlation between Tfh cells and neutralizing antibody responses; however this drawback may be counterbalanced by the critical need for additional studies such as this one, that can provide additional data as well as confirm previous findings.

Part II – Major Issues: Key Experiments Required for Acceptance

Please use this section to detail the key new experiments or modifications of existing experiments that should be absolutely required to validate study conclusions. Generally, there should be no more than 3 such required experiments or major modifications for a "Major

Revision" recommendation. If more than 3 experiments are necessary to validate the study conclusions, then you are encouraged to recommend "Reject".

Reviewer #1: The zebra facs plots gated on pTfh cells in Fig S1A, 2A and 4C are hard to review and should be in pseudocolour or contour plots showing outliers to determine exactly how many events are in the OX40+PDL1+ gate, especially for these rarer cell populations.

PD-1 expression looks quite dim and so the data may be missing some real responses. Instead of gating CXCR5 on SSC and then gating CXCR5+ cells on PD-1 versus SSC, the authors could have analysed CXCR5 against PD-1. This will be more consistent with the literature and it may help with where you put your PD-1+ gate. Does this improve your results??

To show all flow-acquired events, we have converted all flow plots to pseudocolor plots (**Fig 2** & **Fig 4**). Our PD1+ gate was set by a fluorescence minus one (FMO) control; therefore, we prefer gating on CXCR5 then PD1. However, even when gated on CXCR5 versus PD1, we do not observe a significant difference in our results.

The discussion describes a new data figure S4, this needs to be moved into the Results section before it is further explained in the Discussion.

We have moved **S4** (now **S6 Fig**) to the results section (**Lines 250-266**).

Reviewer #2: For the point of this paper, to emphasize potentially novel and meaningful characteristics of the Tfh response specificity and kinetics with integration with the antibody response, a comparison of human Tfh response kinetics to a prototype influenza infection among similarly aged subjects would be very useful.

As mentioned above, we completely agree that a comparison between SARS-CoV-2-induced pTfh responses to those seen in influenza infection is an important future direction of this work; however, we feel that assessing pTfh responses in influenza infection is outside the scope of this paper.

Also, more detailed kinetics of the antibody and CD4 T cell response decay over the longer time of convalescence would be an important and valuable contribution to our understanding of the immunity generated by SARS-CoV-2 infection in humans.

To more fully illustrate the kinetics of the CD4 T-cell and pTfh responses, in a subset of 4 individuals, we screened for response at later timepoints, > 90 days after symptoms onset. We detect several SARS-CoV-2-specific CD4 T-cell responses and one pTfh response at Visit 3. One S protein-specific CD4 T-cell response was even detectable at Visit 4, 190 days after symptom onset. Overall, we observe a contraction of response magnitude (**Figure 5**, **Supplemental Figure 7**).

Characterization of the non-Tfh component in the CD4 T cell response would have been useful to explore.

We agree that is an important question and have added some discussion to this point at **Lines 312-321**. Unfortunately, we feel that characterization of the non-Tfh component beyond the ICS staining is outside the scope of this manuscript.

Reviewer #3:

1. A major strength of this paper is the observation that Tfh responses are extremely robust during the second visit where pTfh were analyzed compared to the first visit. However, the findings would be substantially more clear if there was an additional follow up visit to that pTfh cells in the blood could be assessed, even if done for only a few of the subjects in the study. Do the authors of the study have any additional follow-up data (3rd visit) for even some of the patients that would indicate whether or not the antigne-specific Tfh cell response is maintained, increased, or begins to decrease compared to the second visit? While this may not be possible within the scope of the study design and/or the samples that are already collected, if the data is available to try to address an additional later timepoint it would substantially provide additional kinetics information that would increase the impact of this paper.

This was a great suggestion, and in the four individuals who mounted high magnitude pTfh responses at Visit 2, we were able to assess SARS-CoV-2-specific CD4 T-cell and pTfh responses at Visit 3 and Visit 4 (>90 days after symptom onset, about 60 days after visit 2). Late in convalescence, we detect SARS-CoV-2-specific CD4 T-cell responses in 3 of the 4 individuals tested, with one S protein-specific responses detected 190 days post-symptom onset. Only one pTfh response in one individual was detected at Visit 3. Overall, we observe a contraction of responses (**Fig 5**, **S7 Fig**).

2. One of the major points that is brought up in this study is that there are many patients that are non-responders for their CD4 T cell response. With this conclusion in mind, it would be helpful for the authors to include some discussion about the sensitivity of the AIM assay itself, which according to several previous papers that utilize AIM assay approaches, claim that the assay underestimates the antigen-specific T cell response (to other infections/antigens). This raises the possibility that many of what may be considered non-responders in this study, actually do have antigen-specific CD4 T cell responses, but because of limitations of the assay, these responses are underestimated. A more thorough discussion of these possibilities would be helpful.

In terms of sensitivity of the AIM assay, the majority of studies we have seen has demonstrated that the activation-induced marker expression has a high sensitivity in detecting antigen-specific responses (Reiss 2017 PLoS One, Bowyer 2018 Vaccines). In this publication as well, we have found that AIM is a more sensitive method to detect CD4 responses compared to traditional IFN-γ ICS flow cytometry (**S4 Fig**), likely because AIM detected total antigen-specific CD4 T cells regardless of cytokine production.

Part III – Minor Issues: Editorial and Data Presentation Modifications

Please use this section for editorial suggestions as well as relatively minor modifications of existing data that would enhance clarity.

<u>**Reviewer #1:**</u>

- Line 101: Please add “to” after “previously been shown”

- Fig 2A: Please re-order the FACS plots as M, N, S to keep it consistent and label the donor codes for the representative plots.

- Ref #11 is repeated but updated in Ref #38. Please update.

- For responder frequency graphs, the labels are different for each figure and have versions of responder (Fig 2B) or frequency (Fig 4A, S4D) labelled on the y-axes. Please ensure all of these graphs are labelled consistently with at least the label "Responder frequency" as to not confuse it with the actual data frequencies, which are again interchanging between magnitude and frequency labels.

These four points have been addressed.

Reviewer #2: Better description of methods and control conditions as well as gating strategies would have helped in interpretation of data. Showing more of the primary data from the AIM assay as supplemental data, as well as more information about the controls would have helped interpret the data provided

We have added more description of methods and control conditions. We have also added additional flow staining of pTfh responses in **S4 Fig** and are showing all detected SARS-CoV-2-specific responses at Visit 3 in **S7 Fig**.

Reviewer #3:

1. In lines 248-250, the authors state that perhaps the delay in pTfh cell responses could be related to T cell dysfunction. In this regard, it might be interesting to provide additional discussion as to whether the AIM assay, which already underestimates the antigen-specific response, has additional shortcomings when it comes to measuring T cell dysfunction.

This is a valid point, and we have added additional discussion in **Lines 322-324**.

2. In the discussion section, new and important data are introduced (lines 257-265). As these data are very important for understanding and interpreting the significance of the study, it would be very helpful to move these data into the main Results section.

We now introduce this data (now **S6 Fig**) in the Results section.
